# Functional Diversification within a Predatory Species Flock

**DOI:** 10.1371/journal.pone.0080929

**Published:** 2013-11-21

**Authors:** Edward D. Burress, Alejandro Duarte, Wilson S. Serra, Marcelo Loueiro, Michael M. Gangloff, Lynn Siefferman

**Affiliations:** 1 Department of Biology, Appalachian State University, Boone, North Carolina, United States of America; 2 Sección Zoologia Vertebrados, Departmento de Ecologia y Evolución, Facultad de Ciencias, Montevideo, Uruguay; 3 Sección Ictiologia, Departamento de Zoologia, Museo Nacional de Historia Natural, Montevideo, Uruguay; University of Bologna, Italy

## Abstract

Ecological speciation is well-known from adaptive radiations in cichlid fishes inhabiting lentic ecosystems throughout the African rift valley and Central America. Here, we investigate the ecological and morphological diversification of a recently discovered lotic predatory Neotropical cichlid species flock in subtropical South America. We document morphological and functional diversification using geometric morphometrics, stable C and N isotopes, stomach contents and character evolution. This species flock displays species-specific diets and skull and pharyngeal jaw morphology. Moreover, this lineage appears to have independently evolved away from piscivory multiple times and derived forms are highly specialized morphologically and functionally relative to ancestral states. Ecological speciation played a fundamental role in this radiation and our data reveal novel conditions of ecological speciation including a species flock that evolved: 1) in a piscivorous lineage, 2) under lotic conditions and 3) with pronounced morphological novelties, including hypertrophied lips that appear to have evolved rapidly.

## Introduction

Species flocks are monophyletic assemblages of closely-related species that occur in sympatry and display high degrees of endemism [1] that are often in the process of radiating along an ecological gradient. Although examples of spatially driven (i.e., allopatric) radiations are abundant, recent evidence suggests that ecological selection may play an important role in sympatric speciation events [2-5]. For example, resource segregation is often associated with cases of adaptive divergence among sister species [6,7] and among species flocks [8,9]. 

To date, examples of ecological differentiation among species flocks have been documented primarily in lentic fishes [9-11] and divergence is generally associated with habitat (i.e., pelagic-littoral zonation) heterogeneity. Indeed, ecological speciation in sympatric or parapatric conditions is often tied to habit use. For example, among fishes, deep- or robust-bodied forms are often found in littoral zones while shallow- or slender-bodied forms are associated with pelagic zones [6,13,14]. Pronounced habitat zonation (i.e., depth, temperature, dissolved oxygen) characterizes lentic ecosystems. Such habitat zonation may be limited in lotic environments, where water current seems to be the primary selective agent [15]. No prior studies have documented adaptive radiations of lotic species flocks. 

Sympatric adaptive radiation (i.e., species flocks) is also limited to ecological differentiation among groups with low trophic levels such as algivory, planktivory, zooplanktivory and invertivory [6,8,13]. There are several possible explanations for the rarity of piscivorous lineages producing species flocks. Piscivory tends to limit diversification of feeding structures [16] and thus reduces the likelihood of disruptive ecological selection and niche plasticity. Additionally, fishes tend to be motile, such that habitat zonation (i.e., littoral vs. pelagic) among prey fishes may be an ineffective means of resource partitioning (e.g., source of disruptive selection; [17]) compared to prey types that are associated with a discrete habitat such as plankton (i.e., pelagic zone) or algae (i.e., photosynthetic zone). Furthermore, individual fitness benefits (e.g., increased growth and survivorship; [18]) associated with piscivory may inhibit niche divergence away from piscivory. Such limitations would also constrain the ability for disruptive selection to promote ecological divergence [5]. Thus, piscivorous lineages tend to 1) not evolve away from piscivory and 2) display limited ecological diversification (morphologically or functionally) relative to non-piscivorous lineages [8,19]. 

Recently, in subtropical South America, two species flocks of riverine cichlids (both *Crenicichla*) have been described [15,20]. These assemblages are monophyletic, sympatric at broad and local scales and are endemic to the Uruguay and Paraná rivers [15,20-23], thereby satisfying all species flock criteria [1]. These species flocks include eight (Uruguay) and five (Paraná) species groups within the genus *Crenicichla* that display shallow genetic divergence (<2 My; [15,20]) mirroring many patterns observed in classic species flock examples (e.g., African lake species flocks). *Crenicichla* are also the most speciose cichlid genus [20] and are known to exert strong co-evolutionary pressure upon their prey via direct predation [24]. 

**Figure 1:**
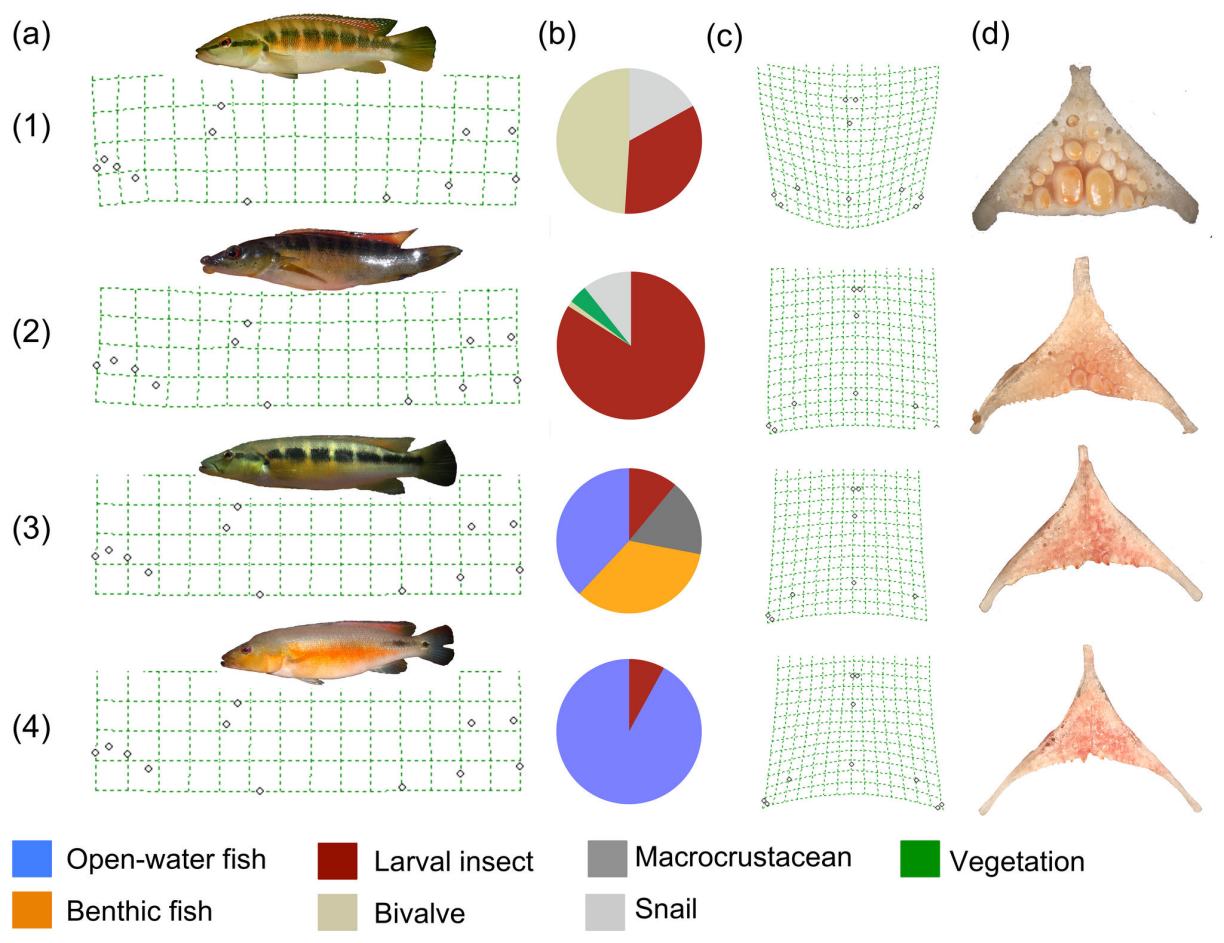
Ecomorphological and dietary comparison of the Uruguay River *Crenicichla* species flock. Live representatives of *C. minuano* (1), *C. tendybaguassu* (2), *C. missioneira* (3), and *Crenicichla celidochilus* (4) are laterally associated with their whole body warp transformation grids (a), summaries of their stomach contents (b), lower pharyngeal jaw warp transformation grids (c) and representative lower pharyngeal jaw (d).

Here, we evaluate ecological divergence in a lotic species flock of cichlid fishes to make inference about potential role of ecology in their radiation. Our objectives were to 1) evaluate morphological divergence of external (whole body) and internal (lower pharyngeal jaw) structures, 2) evaluate trophic divergence using stomach contents and stable isotopes, and 3) reconstruct the evolution of ecological differentiation. We analyzed four members of the Uruguay River *Crenicichla* (URC) species flock: *Crenicichla celidochilus* (CRCE), *C. missioneira* (CRMS), *C. minuano* (CRMN) and *C. tendybaguassu* (CRTE) Lucena & Kullander 1992 (Figure 1); that are sympatrically distributed throughout the drainage and are the least genetically divergent [15,20,21,23]. We chose these species because despite displaying shallow genetic divergence they display obvious morphological disparity, particularly with structures associated with feeding [20]. The sympatric distribution of these species also allows for direct comparison of our metrics, particularly stable isotope analyses that are sensitive to spatial variation. Furthermore, it is established that these species share microhabitats [15,20,21,23], presenting a possible example of adaptive radiation in which spatial divergence is not the primary selective pressure. 

## Methods

### Ethics statement

This research was conducted under Direccion Nacional de Recursos Acuaticos (DINARA) permit 202/1383/2010 and was approved by the Appalachian State University Institutional Animal Care and Use Committee (IACUC) permit #10-07. The Bergós and Sanchis families kindly allowed us to use their land to access study sites. The data presented in this paper are available as Supplementary files (i.e., stomach content analysis) or are already in the public domain (i.e., isotopic ratios of all prey items used in the mixing model; [25,26]). Sequences associated with the phylogeny utilized herein [15] are available at http://www.ncbi.nih.gov/Genbank/ (accession numbers GQ199902-GQ1999963).

### Sampling

We sampled fishes in a ~5 km reach of the Cuareim River (S 30°46, W 056°02) using a backpack electro-fisher, casting nets and hook-and-line in March and November 2010. We froze caudal muscle samples for stable isotope analysis and preserved stomachs for dissection in laboratory. The most abundant potential prey items according to surveys [25,26] were manually sampled for stable isotope analyses. Specifically, we tried to sample appropriate representatives of functional prey groups (Table S1) that may display unique isotopic signatures. For detailed information about the study site and community structure see 25,26. 

### Geometric morphometrics

We used a shape principal component analysis (PCA) of the whole body and lower pharyngeal jaw to investigate biologically meaningful shape differences between species. We used 12 landmarks that describe the shape of the body (Figure S1): CRCE (N=29), CRMS (N=20), CRMN (N=20) and CRTE (N=13) and 10 landmarks that describe the shape of the lower pharyngeal jaw (Figure S2): CRCE (N=9), CRMS (N=10), CRMN (N=10) and CRTE (N=8). Landmark configurations were adapted from [6]. Landmarks were superimposed and aligned by the generalized Procrustes superimposition procedure [31] producing consensus configurations for the whole body and lower pharyngeal jaw. Thin-plate splines were used to calculate interpolation functions (principal warps) among landmarks. Principal component analyses were performed over the partial warp matrices. Uniform component scores were generated by tpsRelw software [32] that represent the most important shape differences among species. Species were identified according to [21]. Voucher specimens are deposited in the Auburn University Museum (Auburn, Alabama, USA), Facultad de Ciencias (Montevideo, Uruguay), and Museo Nacional de Historia Natural de Montevideo (Montevideo, Uruguay).

To quantify species-specific morphology and trophic function, we used our PC scores for the whole body and lower pharyngeal jaw and stable isotope ratios (i.e., niche space) and classified individuals using canonical variate functions (CVA) in SPSS (ver. 20.0; SPSS, Inc., Chicago, IL.). Reclassification success (%) indicates the proportion of individuals that were successfully classified into their pre-designated groups (higher percent ≈ more distinct). This metric incorporates both the importance of neighbor distance (i.e., Euclidean distance) and individual variability (i.e., variation around means). 

### Stomach contents

Stomachs were injected with and preserved in 10% formalin and dissected in laboratory: CRCE (*N*=30), CRMS (*N*=44), CRMN (*N*=37), and CRTE (*N*=26). We identified contents to family level, quantified items using graduated cylinders and calculated percent by volume (%V) and percent occurrence (%O) for each item. Prey items were also organized into functional groups. 

### Stable isotopes

Lyophilized caudal muscle of fishes and whole invertebrates were ground into a homogenous powder, and analyzed for natural abundance stable carbon (δ^13^C) and nitrogen (δ^15^N) isotopes at the Colorado Plateau Stable Isotope Laboratory (Northern Arizona University, Arizona, USA): CRCE (*N*=10), CRMS (*N*=13), CRMN (*N*=18) and CRTE (*N*=5). Isotope values are expressed in parts per million (‰), reflecting their deviation from universal standards: PDB limestone (δ^13^C) and atmospheric nitrogen (δ^15^N). Consumer (e.g., *Crenicichla*) C/N ratios were below 3.5 [25], therefore we did not correct for lipid content (i.e., [27]). A detailed fractionation experiment is beyond the scope of this study. However, to approximate *Crenicichla*-specific fractionation rates, we fed 6 individuals (2 CRMN, 2 CRCE, 2 CRMS) shrimp-based commercial fish food (Omega One Shrimp Pellets, OmegaSea, Ltd.) for 24 months in the laboratory, then prepared caudal muscle and food samples as previously detailed. This time scale is well beyond isotopic turnover estimates according to natural enrichment experiments [28] using ecologically similar fishes (Centrarchidae; [29]). Despite not being able to estimate turnover time/rate (via repeated sampling), our results should accurately reflect *Crenicichla* fractionation rates. 

To estimate the relative (%) assimilation of prey items by consumers we used IsotopeR [30], a dual-isotope (C and N) Bayesian mixing model. We incorporated error associated with 1) our experimental fractionation values (±1.4 and ±0.16 for C and N, respectively), 2) study-wide measurement error (among standards), 3) source concentrations (of C and N) and 4) error associated with sources and consumers into our model. The IsotopeR mixing model assumes that both isotopes are assimilated equally and has limitations associated with source discrimination (reviewed in 30). Sources (e.g., functional prey groups; Table S1) were chosen a priori using stomach content analysis. If a group represented <1% of the stomach contents by volume, it was not included in the mixing model to avoid erroneously attributing prey items to consumers. Because CRCE only consumed two functional prey groups, we included the most feasible third source (benthic fishes) to fulfill the three-source requirement of the model. We employed mixing models to test the relative assimilation of functional groups that were prey items of consumers (via stomach content analyses), not to independently evaluate the diet of *Crenicichla*. Thus we do not consider the two methods entirely independent, however, the conservative exclusion criteria (<1%) should limit circularity. 

### Guild evolution

We reconstructed the evolution of head shape (e.g., prognathus jaws, isognathus jaws, or hypertrophied lips) and trophic guild using maximum likelihood (mk1 model) estimation via a stochastic model using Mesquite 2.75 [33]. In addition to the URC species flock, we included several outgroups (12 total species) to assist in estimating ancestral traits. Head shape characteristics were based on those described in the species’ descriptions [15,22]. Phylogenetic relationships are based on [15]. The tree was pruned to include only species whose trophic guilds were known from the literature (Table S2). We used four discrete variables that describe each species’ diet: ‘generalist’ representing species that consume primarily invertebrates but secondarily may consume fishes, ‘invertivore’ representing species that only consume invertebrates, ‘molluskivore’ consumes primarily mollusks (e.g., snails or bivalves) and secondarily may consume other invertebrates, but does not consume fish and “piscivore” representing species that primarily consume fish. In attempting to resolve branches, we used coarsely defined trophic guilds. For example, CRCE (open-water fish specialist) and CRMS (generalist piscivore) were coded as piscivores. 

## Results

**Figure 2:**
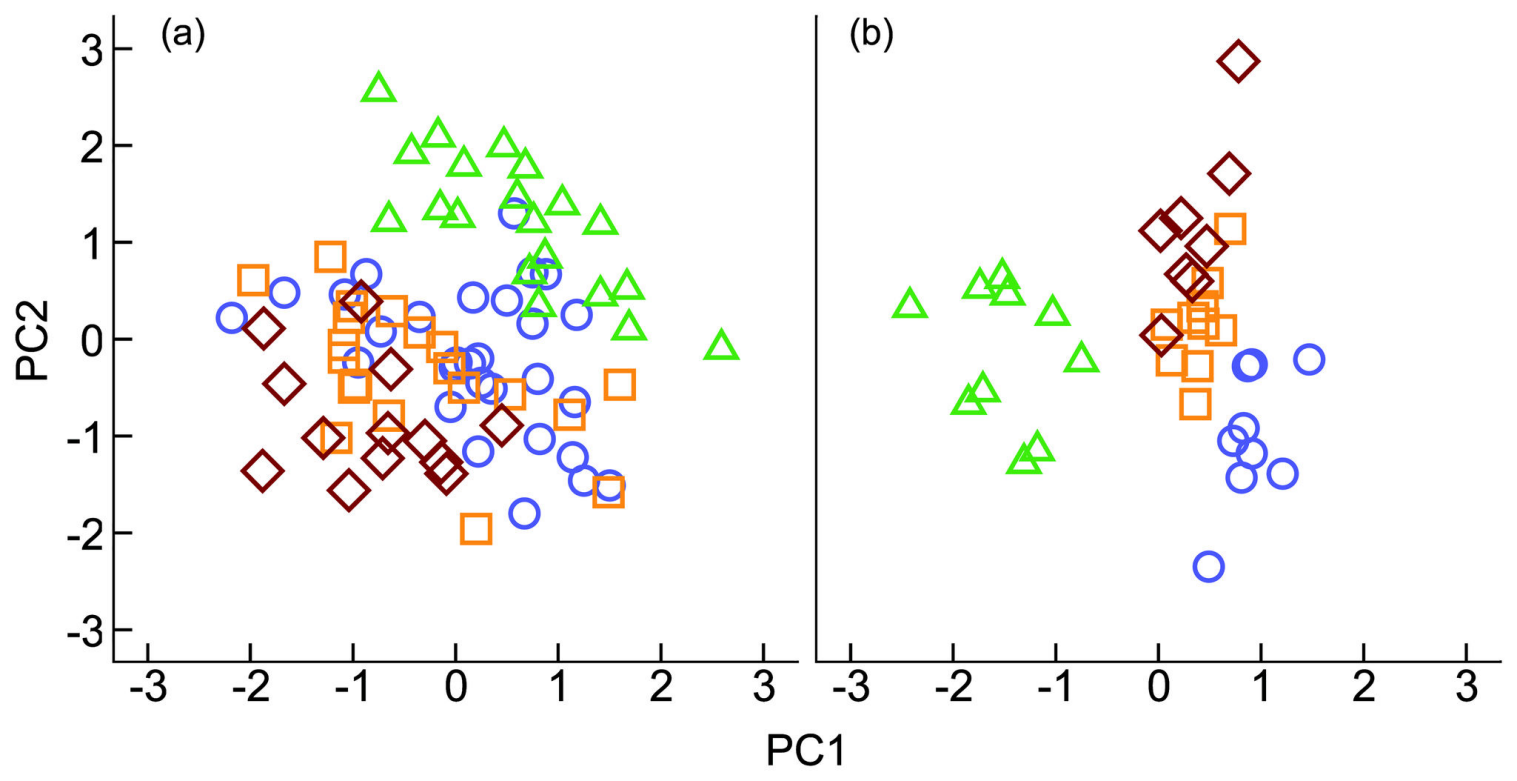
Morphological comparison of Uruguay River *Crenicichla* species flock. Shape principal component analysis of the whole body (a) and lower pharyngeal jaw (b) of *Crenicichla celidochilus* (○), *C. missioneira* (□), *C. tendybaguassu* (◊), and *C. minuano* (△).

**Figure 3:**
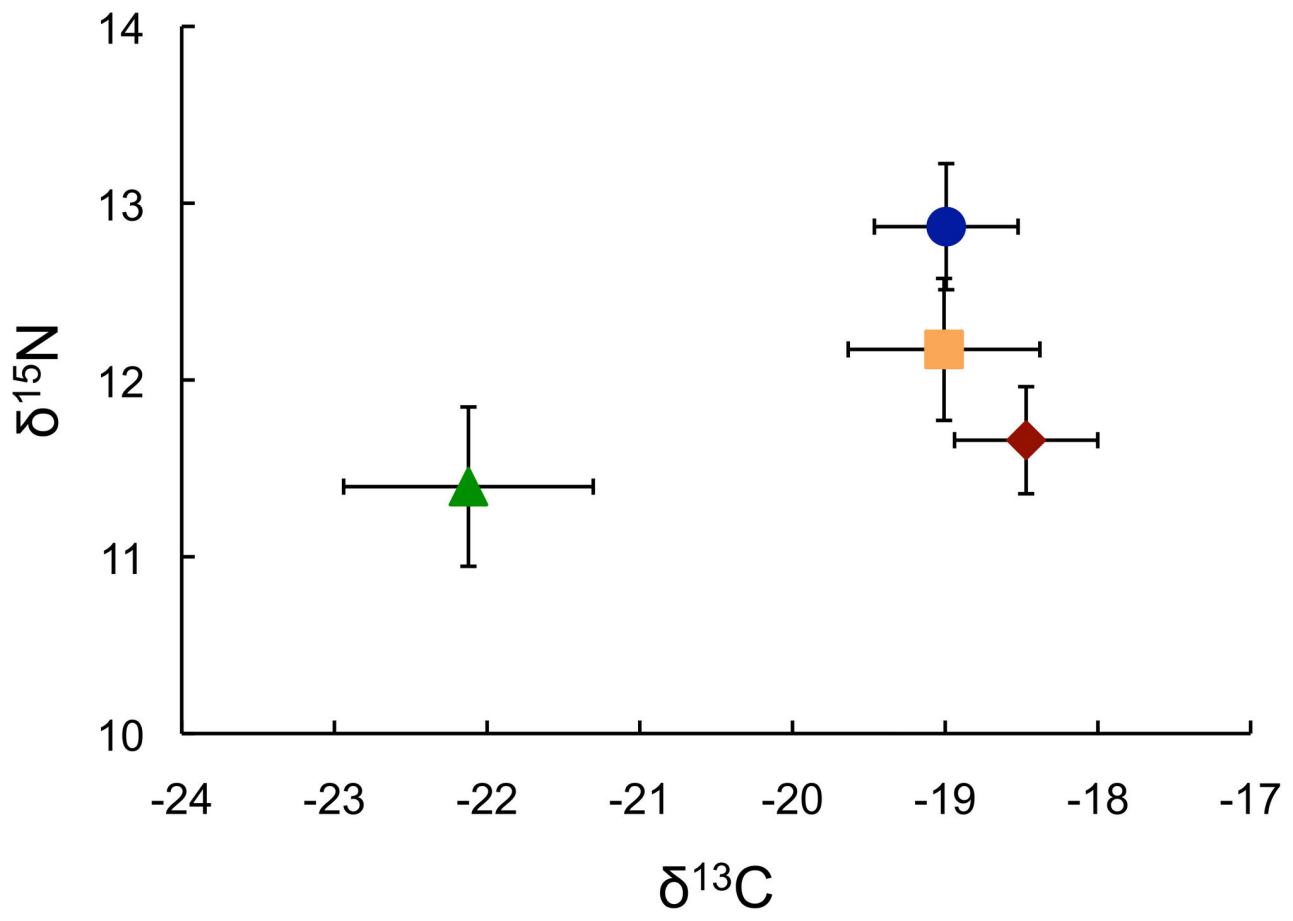
Stable isotope ratios of the Uruguay River *Crenicichla* species flock. Stable nitrogen and carbon isotope ratios (mean±SD) of *Crenicichla celidochilus* (○; CRCE), *C. missioneira* (□; CRMS), *C. tendybaguassu* (◊; CRTE), and *C. minuano* (△; CRMN).

Head shape drives species-specific differences in overall shape (CVA=67.1%; *P*<0.0001; Figures 1 and 2a). For example, head shape varied greatly between species, forming three groups: isognathus jaws (CRMN), prognathous lower jaw (CRCE and CRMS) and jaws with hypertrophied lips (CRTE; Figure 1). Lower pharyngeal jaw shape is highly species-specific (CVA=91.9%, *P*<0.0001; Figures 1 and 2b) and reveals a transitional relationship between CRCE, CRMS and CRTE in shape space that is mirrored by relationships in niche space (Figure 3).

Stomach content analysis revealed that *Crenicichla* diets were species-specific (Table S3; Figure 1). Small benthic invertebrates were the main prey type of CRTE, while CRMS consumed primarily fishes, consisting of both cichlids and characids (Table S3). Two species displayed specialized diets: CRCE consumed almost exclusively characid fishes, and CRMN consumed primarily mollusks, consisting of both bivalves and snails (Table S3). 

Study-wide isotopic error (among standards) was ±0.058 (C) and ±0.107 (N). Stable isotope ratios (CVA=78.3%; *P*<0.0001; Figure 2) were species-specific indicating species derive nutrients from different sources (or in different proportions). Our 24-month fractionation experiment resulted in mean consumer ratios of 12.2±0.2 (N) and -21.7±0.3 (C). We subtracted the food ratios (9.7±0.1 and -23.8±0.2 for N and C, respectively) directly from consumer signatures to determine fractionation rates: 2.5‰ (N) and 2.1‰ (C). Fractionation is dependent on food source C/N ratios [34] such that high and low C/N foods elicit large and small fractionation rates, respectively. The C/N ratio (6.95±0.13) of the shrimp-based pellet food used in our study is similar to ratios of invertebrates consumed by pike cichlids in this system [26]. Additionally, our experiment did not result in atypical consumer isotope ratios (Figure 3), suggesting the shrimp-based pellet food was isotopically and stoichiometrically analogous to natural food resources. Mixing models supported stomach content data such that these species assimilate a large fraction of C and N from prey items that were volumetrically important to their diet (Table 1). The mean contributions of crustaceans and plant material for CRMS and CRTE rounded to zero. Thus, we reran the model with only three sources for both species (Table 1). Snails and benthic fishes may be poorly assimilated, while benthic invertebrates may be preferentially assimilated among these species (Table 1; Figure 1). Furthermore, discrepancies between consumption and assimilation estimations reveal potential conflicts in how behavioral and physiological traits have evolved within the URC species flock. 

**Table 1:** Relative assimilation of functional prey types by *Crenicichla* as estimated by a dual-isotope Bayesian mixing model (IsotopeR).

	Consumer			
Functional prey group	*C. celidochilus*	*C. missioneira*	*C. minuano*	*C. tendybaguassu*
Benthic fishes	<1 (0-1)	<1 (0-1)	--	--
Open-water fish	68 (22-99)	18 (1-37)	--	--
Benthic insects	32 (0-98)	82 (63-100)	66 (39-78)	99 (63-100)
Macrocrustacea	--	--	--	--
Snails	--	--	4 (0-18)	<1 (1-15)
Bivalves	--	--	30 (21-43)	<1 (0-1)

Values indicate the mean (95% CI) of possible mixtures that satisfied mass balance and are rounded to the nearest integer. Contributions that round to zero are denoted as <1.

**Figure 4:**
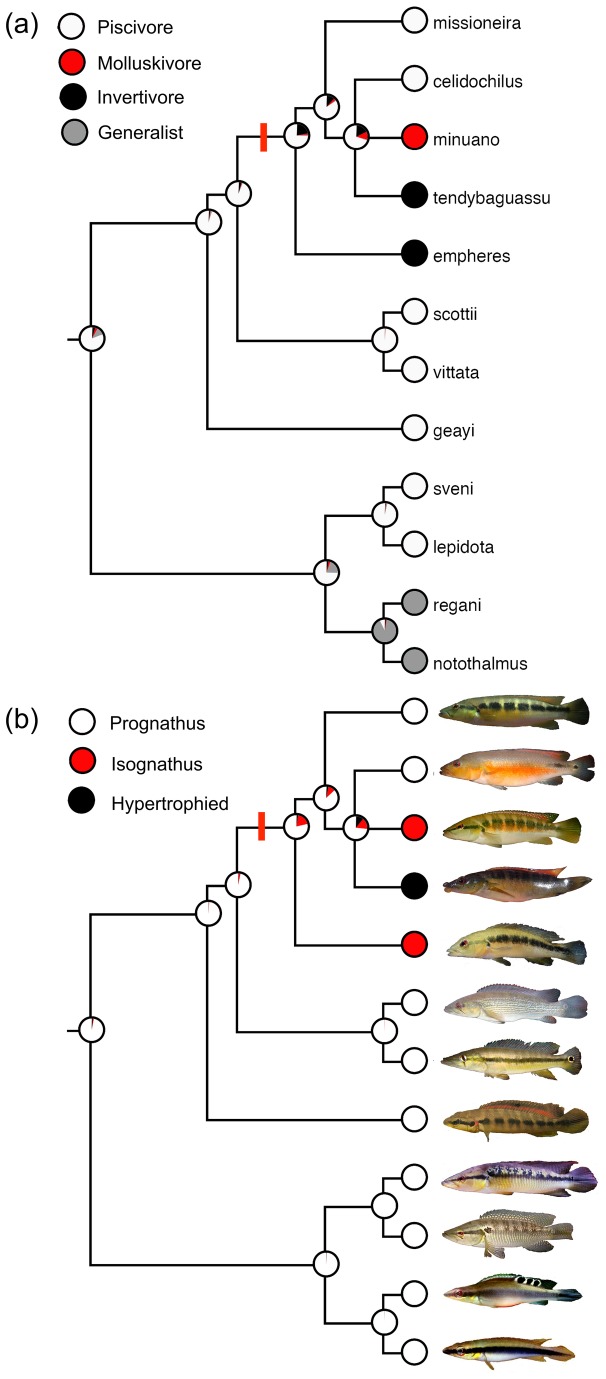
Evolutionary reconstruction of the Uruguay River *Crenicichla* species flock. Maximum likelihood estimation of trophic guild (a) and ancestral jaw structure (b). Phylogenetic relationships are based on [15] pruned to include only species whose diets are known from the literature (see Table S2). Pie diagrams show character states and their proportions (i.e., likelihood) at each node. Maximum likelihood analyses find the ancestral states that maximize the probability that the observed character states (e.g., terminal nodes) would evolve under a stochastic model of evolution [49,50]. Images depict live representatives of each species. Red bar denotes the Uruguay River *Crenicichla* species flock.

Piscivory was resolved as the ancestral trophic state (Figure 4a). This agrees with previous observations of *Crenicichla* [35-38]. Trophic guild evolution within the URC species flock differentiated into four guilds, with molluscivory being unique to the species flock. Our character state analysis also reveals two independent occurrences of divergence from piscivory, and comparatively high trophic diversity among the species flock compared to outgroups (Figure 4a). Prognathus lower jaws were the ancestral state (Figure 4b), which agrees with previous observations of their ubiquity [21]. There are perhaps two independent occurrences of isognathus jaws and only a single occurrence of hypertrophied lips, all occurred with the URC species flock (Figure 4b).

## Discussion

Multiple lines of evidence implicate ecological speciation in the adaptive radiation of the Uruguay River *Crenicichla* (URC) species flock. Despite numerous observations of syntopic foraging among mixed species aggregations [15,20,21,23], species display discrete trophic roles and associated functional morphology. For example, structural novelties such as hypertrophied lips, hypertrophied pharyngeal jaws and at least two evolutionarily independent occurrences of isognathus jaws developed among this highly piscivorous lineage. Additionally, piscivorous ancestral states differentiated into multiple specialized forms (both functionally and morphologically), suggesting the possible importance of competition-based (e.g., disruptive selection) mechanisms in this radiation. Finally, the aforementioned diversification may have occurred rapidly considering the shallow genetic divergence among these species [15,20]. 

Pharyngeal jaw morphology is plastic, strongly correlated with diet and directly reflects functional use (e.g., dietary patterns; [39]). Neotropical cichlids represent multiple speciose adaptive radiations [40] and pharyngeal jaw morphology is often highly co-evolved with trophic function [39] and important components of resource-based ecological partitioning among closely related species [6,41]. Convergent evolution between two lineages with pharyngeal jaws: cichlids and Neartic Centrarchidae [29] suggests that ecological differentiation among such lineages may be predictable. Our data parallel this pattern in the URC species flock. For example, CRCE and CRMS have reduced pharyngeal jaws with sharp teeth necessary for consuming prey whole (e.g., fishes). In contrast, CRMN has hypertrophied pharyngeal jaws with robust molariform teeth necessary for crushing mollusk shells. This finding is consistent with the utilization of hypertrophied pharyngeal jaws among centrarchids and lentic cichlids [41,42].

The URC species flock also differs in fundamental aspects of head shape. Further, this species flock supports several rare morphological traits directly related to trophic function, highlighting the unique trophic diversity of this radiation. *Crenicichla celidochilus* and CRMS have prognathus lower jaws, a ubiquitous state in *Crenicichla* [21], and strongly associated with piscivorous species [37,43]. In contrast, isognathus jaws are an uncommon condition in the genus; yet occur in three species of the URC species flock (CRMN, *C. hadrostigma* and *C. empheres*; [21,22]), including at least two evolutionarily independent occurrences (e.g., CRMN and *C. empheres*). Additionally, although many lentic cichlids display hypertrophied lips [41], they are rare in lotic species (e.g., *Gymnogeophagus*; [44]) and are found in only one of approximately 90 described *Crenicichla* species (CRTE; [21]). Lip hypertrophy is often associated with grazing rocky surfaces and has been associated with other examples of sympatric ecological speciation. For example, thin and hypertrophied lips associated with algivory and invertebrate grazing, respectively, are associated with incipient species in lentic cichlids [41]. Moreover, CRTE consumed many organisms associated with rock surfaces (e.g., Ephemeroptera, etc.) yet we also found that this species frequently consumed detritus-associated organisms (e.g., Chironomidae). Nonetheless, we did not detect any amorphous detritus in CRTE stomachs. This suggests they do not sift substrate and inadvertently ingest large fractions of amorphous detritus like other lotic lineages with hypertrophied lips (i.e., *Gymnogeophagus*; [44]). *Crenicichla tendybaguassu* instead may graze detritus similar to lentic species that graze rocky surfaces [41]. 

Our evidence of adaptive radiation and trophic diversity among a predatory species flock is unique. For example, the URC species flock consumes a wide variety of prey ranging from fishes to benthic invertebrates, bivalves and snails. Most authors consider *Crenicichla* exclusively carnivorous [35-38,43,45,46]. Other putative examples of species flocks occupy low trophic levels; including algivores [8], detritivores [9], benthic sifters and planktivores [6,12,13]. We hypothesize that piscivorous lineages may be less morphologically plastic than lineages feeding more basally and thereby limited in potential niche breadth. For example, the evolution of piscivory limits subsequent morphological diversification [16]. The exact mechanism is unclear, however piscivory may represent a very stable adaptive peak [16], because of the comparatively high selection pressure (e.g., increased survival) among individuals that are capable of exploiting fishes as prey [18]. Thus, it is unique that this lineage has evolved away from piscivory. 

The discrepancies we observed between the relative importance of prey items based on stomach content analysis and stable isotopes reveal potential patterns in how behavioral and physiological traits have evolved within the URC species flock. For example, among the two piscivorous species (CRCE and CRMS), only CRCE assimilate large fractions of C and N from fish. This suggests that the behavioral tendency to exploit fish is decoupled from the ability to efficiently assimilate nutrients from fish. One finding consistent across all four species was that invertebrates were preferentially assimilated relative to the proportions consumed. This finding is intuitive considering that invertebrates are often broken down and assimilated faster than fish [47] due to poorly digestible materials such as bones. Furthermore, plant materials, snails and mollusks were poorly assimilated due to containing relatively higher proportions of carbon compounds (e.g., C/N ratios; [25,26]) that are difficult to digest [34]. We do not believe the discrepancies between stomach contents and stable isotopes affect our character reconstruction, because trophic guilds are conventionally delineated based on consumptive behavior [48] not how nutrients are recycled. However, these findings collectively indicate that among the URC species flock morphological capacity and behavioral tendency to exploit a prey item are decoupled from the physiological ability to preferentially assimilate nutrients from those prey items.

The URC species flock may be unique in that their ecological divergence in not clearly associated with habitat. *Crenicichla* are morphologically constrained to an elongate tubular form, evolved for existence in fluvial conditions [15,21], such that they are not fundamentally adapted to different habitats. Indeed, these species syntopically forage in mixed species aggregations throughout the Uruguay River drainage [15,20,21,23]. This suggests that the ecological differentiation of the URC species flock is not fundamentally linked to habitat use. However, among other species flocks, morphological variation is often vitally associated with habit use. For example, deep- (pelagic) and shallow-bodied (inshore) forms diverged in Nicaraguan crater lakes [6] and large- (inshore) and small-bodied (pelagic) forms diverged in Cameroon crater lakes [13]. Similar robust- and slender-bodied forms have evolved independently multiple times among sticklebacks, which are also associated with littoral and pelagic differentiation [12]. In fact, many low trophic level niches are intrinsically defined by different microhabitats, such as planktivory (pelagic zone) or herbivory (photosynthetic zone). One exception is the Mexican cyprinodont species flock in which divergence does not appear to be associated with habitat usage. This species flock forages heavily upon detritus but partitions subsequently consumed invertebrates [9]. However, disparity in body size was also common among Mexican cyprinodonts, and may explain some resource use patterns. 

Our reconstruction of the evolution of ecological divergence among the URC species flock suggests that piscivorous ancestral states differentiated into multiple specialized forms (both functionally and morphologically) that strongly partition resources. Divergent resource utilization may be indicative of the importance of disruptive ecological selection in adaptive radiations [17]. The URC species flock is sympatric at broad (drainage-wide) and local (mesohabitat) scales [20-23]. The monophyly of this group is supported by recent molecular phylogenies [15,20] as well as morphological and phenotypic synapomorphies [15,21]. Coupled with their shallow genetic divergence [15,20], these factors suggest that the URC species flock may have evolved in sympatric conditions. However, it is impossible to discount the possibility that species distributions (i.e., their sympatry) have changed during their radiation, thus we cannot reject the hypothesis that the URC species flock evolved in allopatric conditions. Nevertheless, we demonstrate that trophic-based ecological divergence was a fundamental component of the diversification among the URC species flock. 

## Supporting Information

Figure S1 **Landmark configuration used to analyze biologically meaningful shape changes in whole body shape.**
(TIF)

Figure S2 **Landmark configuration used to analyze shape variation among the lower pharyngeal jaw.**
(TIF)

Table S1 **Species whose stable isotope ratios were used to delineate functional prey groups loaded into a dual isotope (**C** and **N**) mixing model.** If multiple taxa are listed, they were pooled.
(DOCX)

Table S2 **References used for trophic guild assignment.**
(DOCX)

Table S3 **Stomach content analyses (% by volume / % occurrence) for *Crenicichla celidochilus*, *C. missioneira*, *C. minuano* and *C. tendybaguassu*.** Copepods, cladocerans, and ostracods are pooled under microcrustacea. Vascular plants and periphyton are pooled under vegetation. Items representing 0.1-0.99% by volume are indicated <1. Items representing < 0.1% by volume are indicated <<1. Primary items are highlighted in gray and unique prey items not consumed by other *Crenicichla* are indicated in bold.
(DOCX)
