# Supplementary figures and images for: Functional Diversification within a Predatory Species Flock
Source: PLoS One. 2013 Nov 21;8(11):e80929. doi: 10.1371/journal.pone.0080929 (PMC3836755; doi:10.1371/journal.pone.0080929)

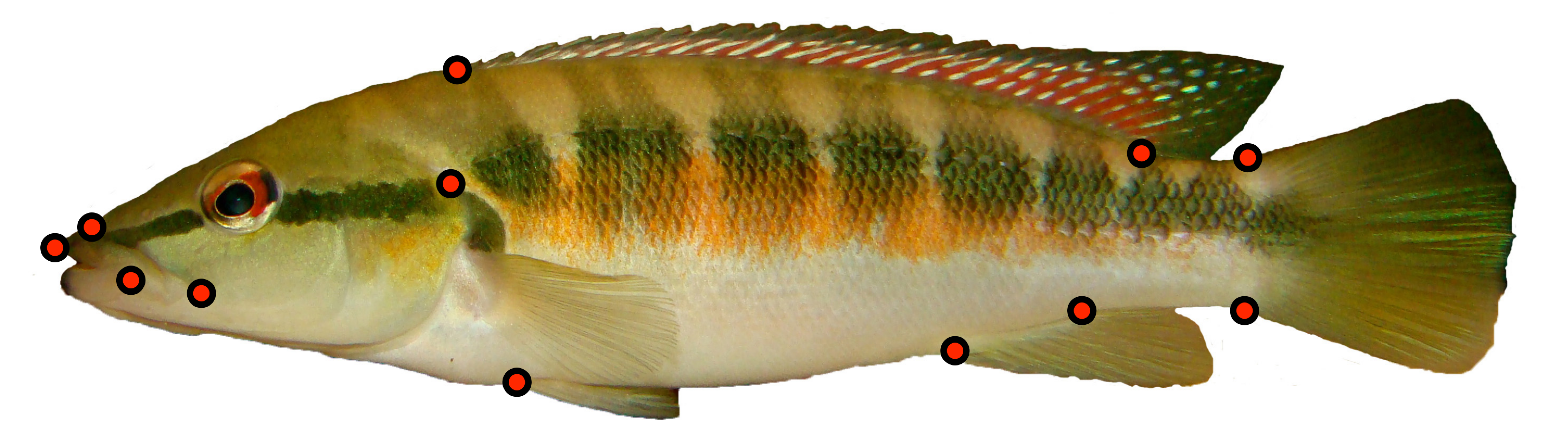

Supplement: Figure S1 — Landmark configuration used to analyze biologically meaningful shape changes in whole body shape. (TIF) [file pone.0080929.s001.tif]

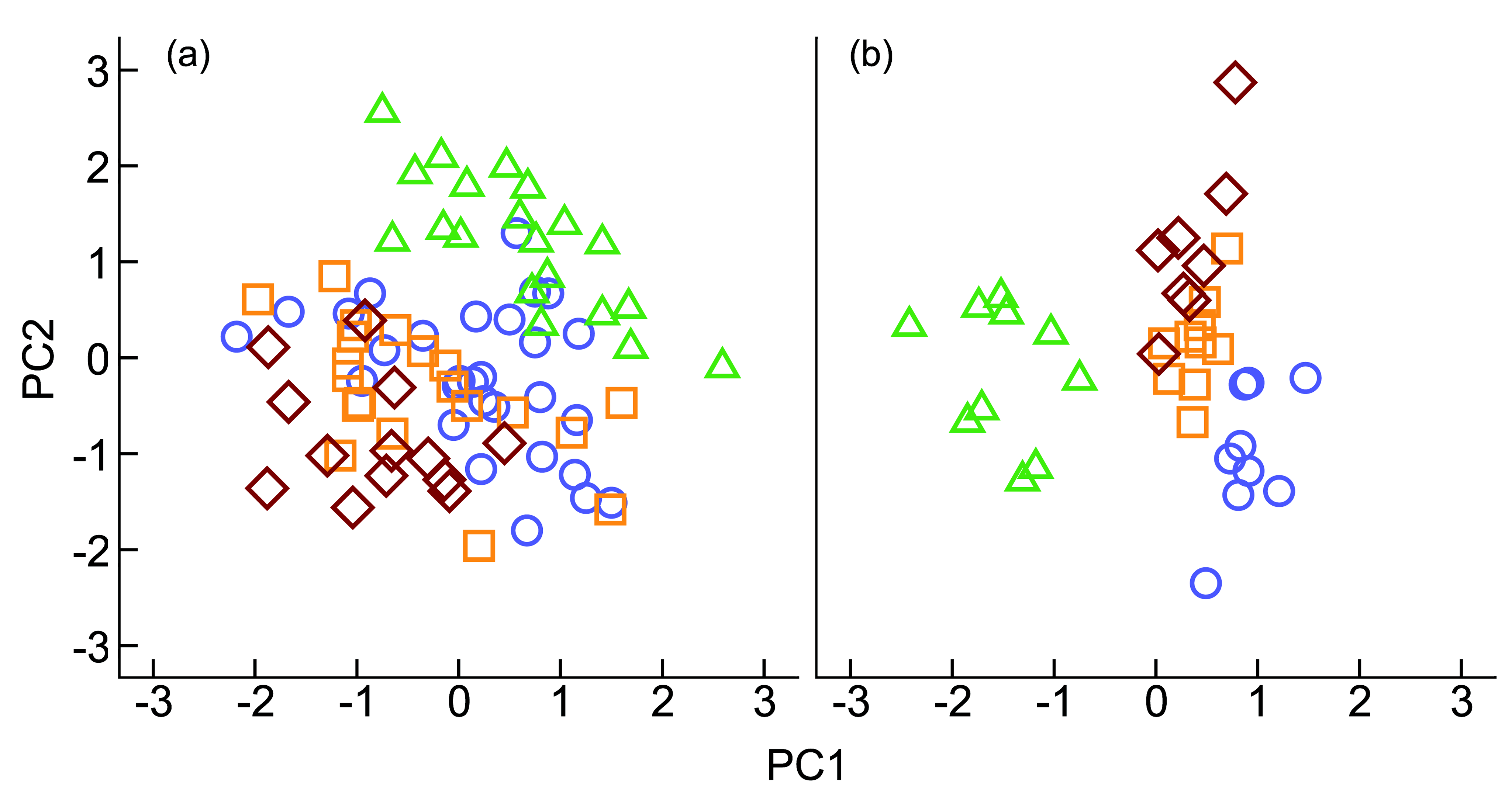

Supplement: Figure S2 — Landmark configuration used to analyze shape variation among the lower pharyngeal jaw. (TIF) [file pone.0080929.s002.tif]
