# Supplementary material for: Functional Diversification within a Predatory Species Flock
Source: PLoS One. 2013 Nov 21;8(11):e80929. doi: 10.1371/journal.pone.0080929 (PMC3836755; doi:10.1371/journal.pone.0080929)
Supplement: Table S1 — Species whose stable isotope ratios were used to delineate functional prey groups loaded into a dual isotope (C and N) mixing model. If multiple taxa are listed, they were pooled. (DOCX) [file pone.0080929.s003.docx]

| Functional prey group | N | Taxa included | Consumers attributed to |
| --- | --- | --- | --- |
| Benthic fish | 9 | *Gymnogeophagus gymnogenys* | CRMS |
| Open-water fish | 11 | *Astyanax* sp. | CRMS, CRCE |
| Benthic insects | 16 | Leptophlebiidae | CRMIS, CRTE, CRMN, CRTE |
| Macrocrustacea | 14 | *Trichodactylus panoplus* | CRMS |
| Bivalve | 5 | *Neocorbicula limosa*, *Corbicula fluminea* | CRMN, CRTE |
| Snail | 9 | Cochliopidae | CRMN, CRTE |
| Plant material | 3 | *Hydrilla* | CRTE |
