# Supplementary material for: Functional Diversification within a Predatory Species Flock
Source: PLoS One. 2013 Nov 21;8(11):e80929. doi: 10.1371/journal.pone.0080929 (PMC3836755; doi:10.1371/journal.pone.0080929)
Supplement: Table S2 — References used for trophic guild assignment. (DOCX) [file pone.0080929.s004.docx]

| Taxa | Trophic Guild | Reference |
| --- | --- | --- |
| *Crenicichla celidochilus* | Piscivore | This Study |
| *Crenicichla missioneira* | Piscivore | This Study |
| *Crenicichla minuano* | Molluscivore | This Study |
| *Crenicichla tendybaguassu* | Insectivore | This Study |
| *Crenicichla lepidota* | Piscivore | 38 |
| *Crenicichla scottii* | Piscivore | 38 |
| *Crenicichla empheres* | Insectivore | 21 |
| *Crenicichla vittata* | Piscivore | 21 |
| *Crenicichla geayi* | Piscivore | 45 |
| *Crenicichla sveni* | Piscivore | 45 |
| *Crenicichla regani* | Generalist | 35 |
| *Crenicichla notothalmus* | Generalist | 46 |
