# Supplementary material for: Functional Diversification within a Predatory Species Flock
Source: PLoS One. 2013 Nov 21;8(11):e80929. doi: 10.1371/journal.pone.0080929 (PMC3836755; doi:10.1371/journal.pone.0080929)
Supplement: Table S3 — Stomach content analyses (% by volume / % occurrence) for Crenicichla celidochilus, C. missioneira, C. minuano and C. tendybaguassu. Copepods, cladocerans, and ostracods are pooled under microcrustacea. Vascular plants and periphyton are pooled under vegetation. Items representing 0.1-0.99% by volume are indicated <1. Items representing < 0.1% by volume are indicated <<1. Primary items are highlighted in gray and unique prey items not consumed by other Crenicichla are indicated in bold. (DOCX) [file pone.0080929.s005.docx]

| Taxa | *C. celidochilus* | *C. missioneira* | *C. minuano* | *C. tendybaguassu* |
| --- | --- | --- | --- | --- |
|  | n=30 | n=44 | n=37 | n=26 |
| Fishes |  |  |  |  |
| Auchenipteridae |  | **<1 / 3.2** |  |  |
| Characidae | 90.6 / 35 | 37.7 / 48.4 |  |  |
| Cichlidae | <1 / 45 | 33.9 / 35.5 | <1 / 10.5 |  |
| Loricariidae | **<1 / 5.0** |  |  |  |
| Unidentified | <1 / 15 | 4.6 / 9.7 |  |  |
| Invertebrates |  |  |  |  |
| Bivalvia |  |  |  |  |
| Corbiculidae |  |  | 48.1 / 26.3 | 1.2 / 9.5 |
| Hyriidae |  |  | **<<1 / 5.3** |  |
| Chelicerata |  |  |  |  |
| Acari | < 1 / 25.0 |  |  | 1.4 / 43.0 |
| Lycosidae | **< 1 / 5.0** |  |  |  |
| Coleoptera |  |  |  |  |
| Dytiscidae | << 1 / 5.0 |  |  |  |
| Hydrophilidae | << 1 / 5.0 |  |  |  |
| Psephenidae | << 1 / 5.0 | << 1 / 5.0 |  | <<1 / 9.5 |
| Decapoda |  |  |  |  |
| Aeglidae |  | **<1 / 6.4** |  |  |
| Microcrustacea |  |  | **<1 / 21.1** |  |
| Trichodactylidae |  | **16.9 / 6.5** |  |  |
| Diptera |  |  |  |  |
| Chironomidae | <1 / 20 |  | 6.9 / 7.8 | 7.9 / 85.7 |
| Ephemeroptera |  |  |  |  |
| Baetidae | 6.3 / 40 | <1 / 41.9 | 6.9 / 68.4 | 23.3 / 71.4 |
| Caenidae | <1 / 42.1 |  | 11.4 / 42.1 | 1.9 / 23.8 |
| Leptophlebiidae |  | 3.6 / 48.4 |  | 34.9 / 90.5 |
| Unidentified |  |  |  | 2.6 / 9.5 |
| Gastropoda |  |  |  |  |
| Ampularidae |  |  | 6.7 / 36.8 | 10.7 / 42.9 |
| Ansylidae |  |  | **6.9 / 42.1** |  |
| Cochliopidae |  |  | **3.1 / 26.3** |  |
| Hymenoptera |  |  |  |  |
| Formicidae | **< 1 / 5.0** |  |  |  |
| Odonata |  |  |  |  |
| Gomphidae |  | **1.8 / 6.5** |  |  |
| Trichoptera |  |  |  |  |
| Hydropsychidae |  |  |  | **3.9 / 19.1** |
| Leptoceridae |  | <1 / 16.1 | 7.4 / 68.4 |  |
| Unidentified |  |  |  | 1.8 / 47.6 |
| Vegetation |  |  |  | **4.1 / 38.1** |
